# A new small molecule inhibitor of soluble guanylate cyclase

**DOI:** 10.1016/j.bmc.2015.07.074

**Published:** 2015-09-01

**Authors:** Filipa Mota, Paul Gane, Kathryn Hampden-Smith, Charles K. Allerston, John Garthwaite, David L. Selwood

**Affiliations:** a The Wolfson Institute for Biomedical Research, University College London, Gower Street, London WC1E 6BT, United Kingdom; b Structural Genomics Consortium, University of Oxford, Roosevelt Drive, Oxford OX3 7DQ, United Kingdom

**Keywords:** sGC (soluble guanylate cyclase), Nitric oxide, ODQ, Surface plasmon resonance

## Abstract

Soluble guanylate cyclase (sGC) is a haem containing enzyme that regulates cardiovascular homeostasis and multiple mechanisms in the central and peripheral nervous system. Commonly used inhibitors of sGC activity act through oxidation of the haem moiety, however they also bind haemoglobin and this limits their bioavailability for in vivo studies. We have discovered a new class of small molecule inhibitors of sGC and have characterised a compound designated **D12** (compound **10**) which binds to the catalytic domain of the enzyme with a *K*_D_ of 11 μM in a SPR assay.

## Graphical abstract

**Figure:**
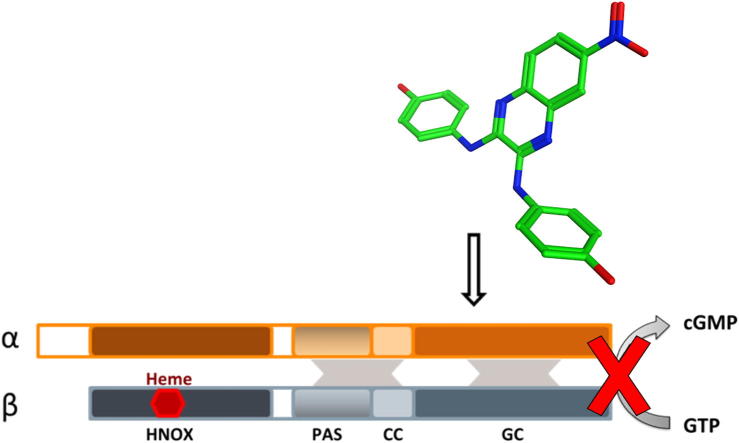

## 1 Introduction

The heterodimeric enzyme soluble guanylate cyclase (sGC) is an endogenous receptor for nitric oxide (NO). NO binds to a haem prosthetic group resulting in a conformational change which activates the enzyme. Upon activation, sGC converts guanosine triphosphate (GTP) into cyclic guanosine monophosphate (cGMP). NO-induced activation of sGC is key to maintaining cardiovascular homeostasis and in the brain, NO—sGC acts as a neurotransmitter—receptor system.^1–3^

NO-induced signalling has been implicated in the modulation of synaptic transmission and to act in long-term potentiation, one of the major cellular mechanisms that underlie the processes of learning and memory.^4^ In rats, high cGMP levels promote neural stem cells differentiation to neurons whilst reduced cGMP levels promote differentiation to non-neuronal (mainly glial) cells, which consequently leads to impaired cognitive function.^5^

**Figure 1:**
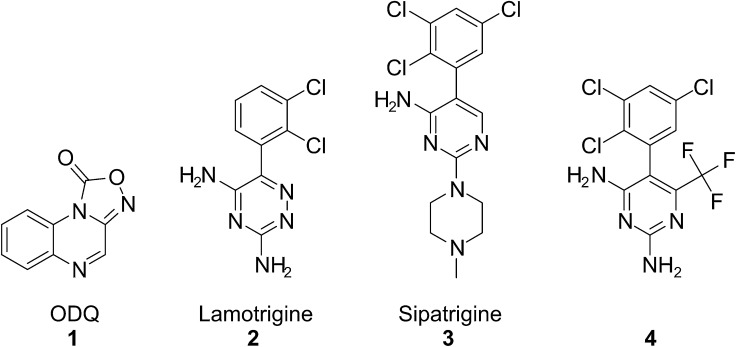
Chemical structures of sGC inhibitor ODQ **1**, Lamotrigine **2**, and analogues Sipatrigine **3** and **4**.

NO can mediate neurotoxicity and cause neuronal cell death. The rapid on–off-kinetics and desensitization profile of NO, combined with variations in the rate of cGMP breakdown, provide fundamental mechanisms for shaping cellular cGMP responses and is important in decoding NO signals under physiological and pathological conditions.^6^ The neurotransmitter is associated with pathogenic mechanisms involved in multiple neurodegenerative diseases, including Parkinson’s Disease (PD).^7^ The NO—sGC system is also involved in the etiology of migraine.^8^ Recent research has suggested the involvement of NO in PD is due to activation of sGC. Disruption of striatal NO-sGC-cGMP signalling cascades resulted in profound changes in behavioural, electrophysiological, and molecular responses to pharmacological manipulations of dopamine and glutamate transmission.^9^ Studies performed in animal models of PD with a sGC inhibitor, ODQ **1** (Fig. 1), have shown that the enzyme could be a new drug target for restoring basal ganglia dysfunction and attenuating motor symptoms associated with PD.^9^ ODQ **1** has been widely used to study the function of the NO-sGC-cGMP signal transduction pathway and it has been a valuable tool to distinguish signalling events mediated by sGC from those involving other nucleotide cyclases.^10^ The small molecule binds to the ferrous haem in the β-subunit of the enzyme, yielding ferric haem which cannot bind NO.^11,12^ Haem-binding compounds such as ODQ **1** and its 8-bromo-analogue NS2028, show activity against other haem containing proteins as such as haemoglobin, albeit at high concentrations.^13,14^ ODQ may also not be able to block cGMP signalling in all circumstances.^15^ Other known ways of inhibiting sGC activity include block of the catalytic site with ATP and GTP analogues^16–20^ though these have weak inhibitory potency. Inhibitors such as LY-83583 act indirectly by generating superoxide which reacts rapidly with NO.^16^ Previously we demonstrated that surface plasmon resonance (SPR) allied to biochemical screening was an effective way of discovering new sGC ligands. In this study we identified a new small molecule inhibitor of sGC, which does not act through oxidation of the haem.

## 2 Results

### 2.1 In silico similarity searching and screening

In the course of studies in our laboratory we observed activation of sGC with the anti-epileptic drug lamotrigine **2** and inhibition with lamotrigine analogues sipatrigine **3** and **4**, all at low millimolar concentrations (data not shown) (Fig. 1). These compounds were used as the starting point for a screening study to find new inhibitors of sGC. The strategy was to conduct several rounds of similarity searching and screening to identify the best inhibitors. Some synthetic studies were conducted on the best hit to explore the structure–activity relationships.

**Figure 2:**
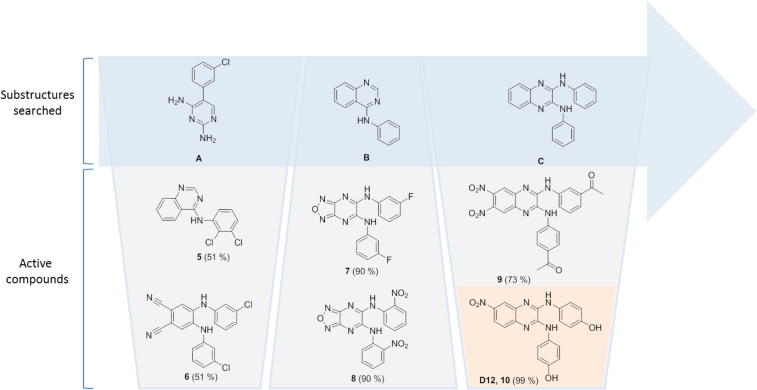
Virtual screening. Chemical structures of virtually searched substructures A–C and the most active compounds **5**–**10** of each series. The values in brackets correspond to the % inhibition of sGC activity at 100 μM compound.

Virtual screening was performed by similarity searches using MACCS fingerprints at 85% and 75% Tanimoto of commercial libraries and the selected compounds were screened at 100 μM against purified bovine lung sGC using diethylamine NONOate (30 nM) as the NO donor. Enzyme activity was determined by measuring cGMP production using a standard cGMP [^3^H] radioimmunoassay.^21,22^ The initial structure searched (Fig. 2, substructure A) resulted in circa 500 structures, out of which 16 compounds were selected based upon diversity, molecular size, and availability. Compounds **5** and **6** showed inhibition of enzyme activity by 51% at 100 μM. Subsequent searches of substructures B and C resulted in the identification of [1,2,5]oxadiazolo[3,4-*b*]pyrazines **7** and **8**, and *N*2,*N*3-diphenylquinoxaline-2,3-diamines **9** and **10** (designated **D12**) as inhibitors of sGC (Fig. 2).

### 2.2 Chemistry and structure activity studies

The hit compound **10** is formed of a quinoxaline scaffold with a nitro group in the 6-position of the heterocycle, and joined to two phenols via secondary amine linkers. A small set of analogues was synthesised to explore the binding role of the substituents, focusing on the nitro group and the phenols (Table 1).

**Scheme 1:**
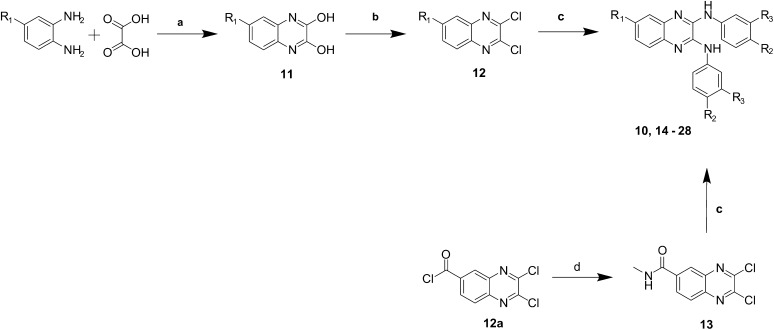
General synthetic route to compounds **10** and **14**–**29**. (a) 4 N HCl, reflux, 2 h; (b) SOCl_2_, DMF, reflux, 2 h; (c) aniline, NMP, 150 °C, 5 min, μW; (d) CH_3_NH_2_, EtOH, DCM, rt, 24 h.

Compound **10** and analogues **14**–**28** were synthesised via nucleophilic aromatic displacement using commercially available anilines and 2,3-dichloro-quinoxalines **12** when possible. In other cases 2,3-dichloroquinoxalines **12** were obtained via a known two-step synthesis, starting with the condensation of 1,2-diamines with oxalic acid.^23^ The resulting 2,3-dihydroxyquinoxalines **11** were chlorinated with thionyl chloride and a catalytic amount of DMF. The carboxamide substituted quinoxaline **13** was obtained after amidation of the carbonyl chloride intermediate **12a** (Scheme 1).

**Scheme 2:**
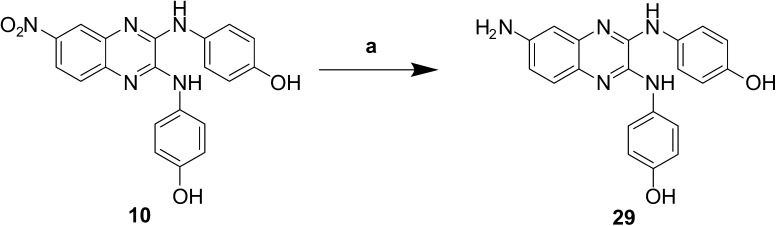
Reduction of nitro group to a primary amine. (a) NaBH_4_, SnCl_2_·2H_2_O, EtOH, reflux, 2 h.

Reduction of the nitro group in compound **10** was successfully achieved using tin(II) chloride in the presence of sodium borohydride to give compound **29** (Scheme 2).

We have previously described a surface plasmon resonance-based assay for the detection of binding of small molecules to full-length sGC and a smaller construct of the catalytic domain (sGCcat).^24^ This assay was used to measure the binding of the compounds to sGCcat.^24^ In general, compounds with hydroxyls on the anilino phenyl rings (**10**, **14**, **20**, **21**, **23**–**26**, **28**, **29**) showed higher binding to the enzyme, suggesting they might be involved in hydrogen bonding. The position of the hydroxyl group can be changed whilst retaining binding strength and activity. However, other groups such as acetamide **17**, **18**, methoxy **15**, or fluoro **19**, rendered the compound biochemically inactive and reduced its binding strength.

The electron-withdrawing nitro group commonly presents as a challenge in drug design. Nitro-aromatics are commonly associated with toxicity, but identifying suitable replacements has proven difficult.^25^ A series of 6-substituted-2,3-dichloroquinoxalines was synthesised. Further modifications included the reduction of the nitro group to a primary amine, and the conversion of an acyl chloride into an amide (Schemes 1 and 2). Changes in the 6-position of the quinoxaline have resulted in compounds which show high or medium binding to the receptor, but poor or null inhibition of its activity, with the exception of the trifluoromethyl compounds **21** and **22**. Removal of the nitro group (compound **20**) showed only a small decrease in binding, but showed no significant inhibition of the enzyme. This would suggest that binding of the compounds is not greatly influenced by the group in the 6-position of the quinoxaline, but this is however required for activity. Insertion of amine **29**, chloro **25**, **26**, and nitrile **23**, **24** groups also resulted in compounds which bind to the receptor but do not inhibit the enzyme activity.

### 2.3 Molecular modelling

**Figure 3:**
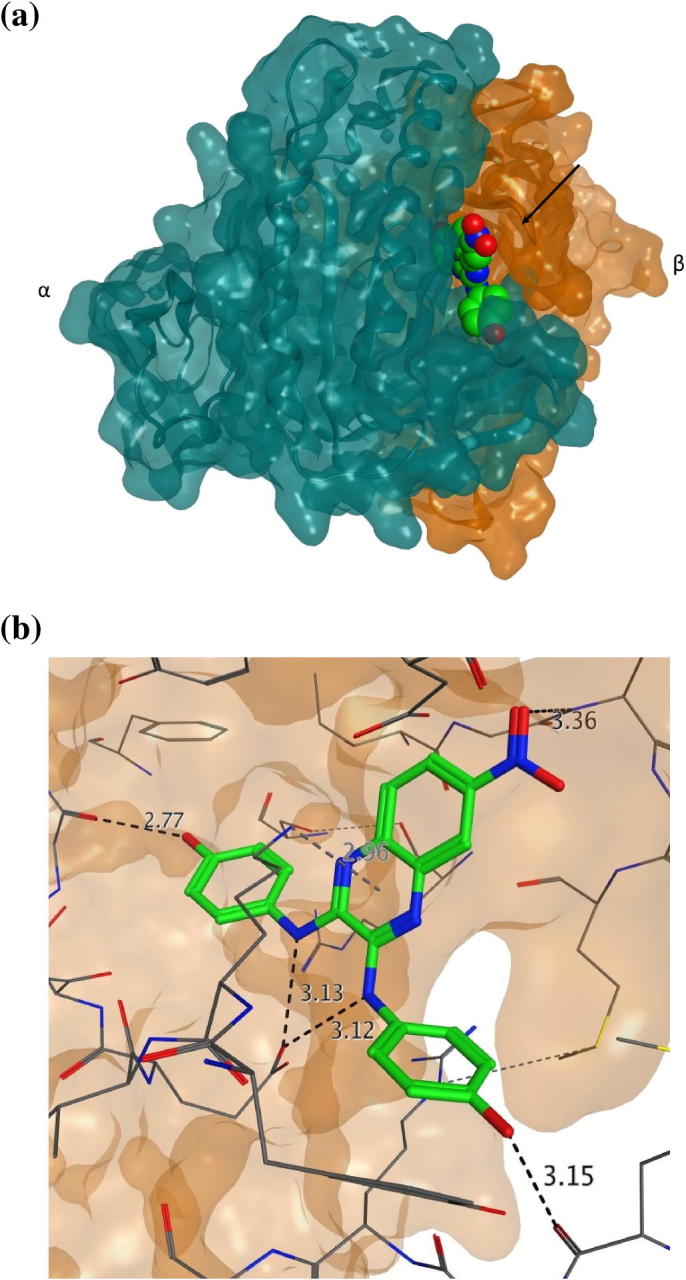
Docking of compound **10** into the allosteric binding site of sGC. (a) Cartoon representation of compound **10** docked at the interface of the two subunits of the catalytic domain of sGC (PDB code 3uvj), in which the α-subunit is coloured green and the β-subunit coloured orange; (b) representation of the ligand interaction between the compound and the residues of sGC.

A possible binding mode was investigated by molecular modelling, using the available crystal structure of sGCcat (pdb code 3uvj). Compound **10** was docked into the allosteric site of sGC and possible binding modes were analysed based on their Goldscore fitness. Most binding modes are similarly docked at the interface of the two subunits. The docking poses show four key interactions with the receptor: one oxygen of the nitro group may act as H-bond acceptor for two backbone NHs, being the only interaction with residues of the β-subunit; the two NH groups attached to the quinoxaline may act as H-bond donors for the side chain of a glutamic acid; one or two OH groups can act as a H-bond donors; and a π–cation interaction is suggested between a charged lysine and one of the aromatic rings. In addition, the lysine and arginine residues present in the site could show π–cation interactions with other aromatic rings of the ligand (Fig. 3).

### 2.4 Biological and biophysical characterisation of compound **10**

**Figure 4:**
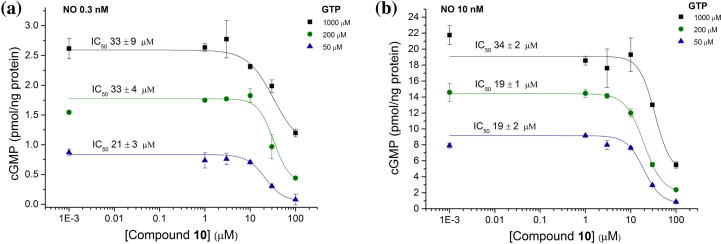
Biochemical characterisation of compound **10** activity. Compound **10** inhibited NO-stimulated cGMP production by isolated sGC with varying concentrations of GTP (50, 200, 1000 μM) and clamped NO concentrations at (a) 0.3 nM and (b) 10 nM.

The activity of compound **10** on sGC was tested in the presence of increasing concentrations of GTP (Fig. 4a) and NO (Fig. 4b). Concentration–response curves did not show appreciable variation amongst different conditions, suggesting activity of the compound is not through competition with NO or GTP. Activation of sGC can also be induced by the haem-mimetic drug cinaciguat in the absence of NO.^26^ Cinaciguat binds to the haem-deficient protein, simulating the NO-activated state.

Compound **10** was capable of inhibiting cGMP production after enzyme activation with cinaciguat, further demonstrating that its mechanism of action is not NO-dependent. Activity against membrane-bound GCs was measured using purified rat lung membranes in the presence of 10 μM ODQ **1** to inhibit NO-activated sGC. cGMP generation was measured after stimulation with atrial natriuretic peptide and compound **10** showed inhibition of pGC-generated cGMP, indicating an action through the catalytic domain of the enzyme (Table 2).

**Figure 5:**
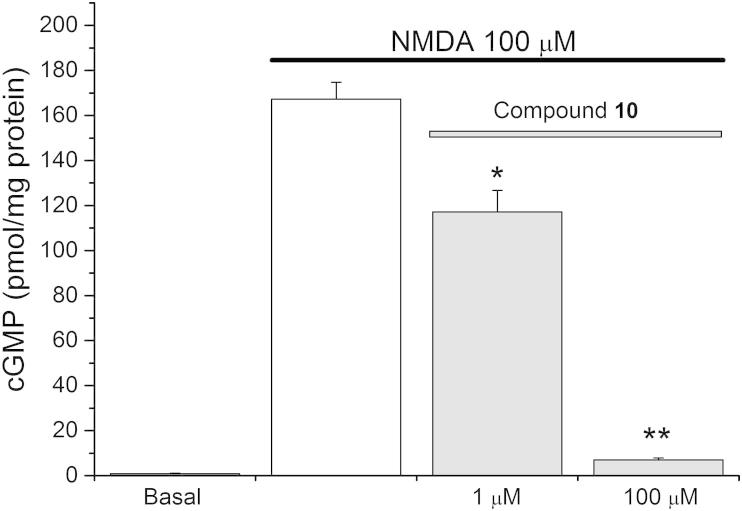
Activity of compound **10** in rat cerebellum. Compound **10** inhibits cGMP production induce by NMDA in rat cerebellar slices at 100 μM. Results are means ± SEM (*n* = 3); ^*^p <0.02, ^**^p <0.001 versus NMDA control (2-sample *t*-test).

The ability of compound **10** to inhibit sGC in tissues was explored using NMDA-stimulated cGMP production in cerebellar slices, which depends on NO formation. Rat cerebellar slices were pre-incubated with compound **10** then treated with NMDA. Compound **10** inhibited the ensuing cGMP generation almost completely at 100 μM and to a lesser extent at 1 μM (Fig. 5).

**Figure 6:**
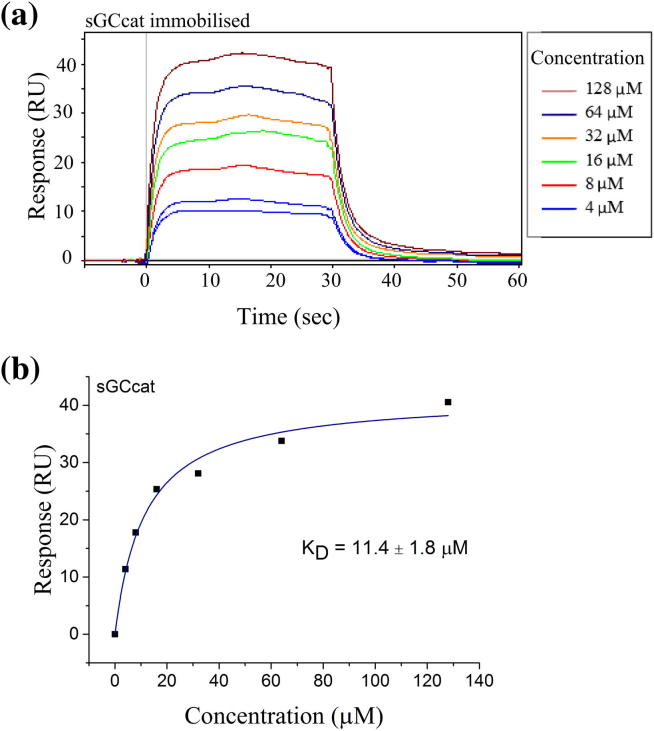
Binding of compound **10** to sGCcat. Compound **10** binds to the catalytic domain of sGC (*K*_D_ = 11.4 ± 1.8). (a) SPR sensorgram and (b) dose–response of compound **10** binding to sGCcat.

The binding affinity of compound **10** to sGCcat was explored in detail using SPR, and suggests a 1:1 interaction between the compound and the dimer, with a calculated *K*_D_ of 11.4 ± 1.8 μM (Fig. 6). Further characterisation with full-length enzyme gave a *K*_D_ of 19.4 ± 2.1 μM.

## 3 Discussion

The sGC heterodimer is composed of a α-subunit and a shorter β-subunit which presents in the N-terminal the prosthetic haem group bound to histidine-105.^27^ Each subunit is composed of a haem-nitric oxide binding domain, a PAS-like domain, a coiled-coil bundle, and the C-terminal catalytic domain where turnover of GTP into cGMP occurs.^28^ The catalytic domain of sGC shows two sites at the interface of the α- and β-subunits that can accommodate small molecules: the GTP binding site, and an allosteric regulatory binding site.^29–31^ The two sites are different, and small molecules can bind to one or to both sites, thus showing a 1:1 or 1:2 binding ratio. We have previously shown by surface plasmon resonance (SPR) that ATP binds to both sites and may inhibit sGC activity via competition with GTP, highlighting the potential for allosteric regulation of the enzyme.^24^ GC domains are also present in the membrane-bound natriuretic peptide receptors type-A and type-B. There receptors, also referred to as particulate GCs (pGCs), are activated by binding of natriuretic peptides to the extracellular domain of the receptors, leading to an increase in GTP turnover at the intracellular GC domain.^32–34^ In this study we observed by SPR that compound **10** bound to the catalytic domain of sGC in a 1:1 ratio. Inhibition of particulate GC was also observed and supported the hypothesis of interaction at the catalytic domain. Binding of compound **10** to an allosteric site that is also capable of binding ATP could suggest these compounds might also act on other ATP- and cyclic-nucleotide—binding domains. We tested compound **10** against adenylyl cyclase, an enzyme that converts ATP into cAMP, and it showed no activity on basal or forskolin-stimulated adenylyl cyclase (data not shown). However, activity against kinases or phosphodiesterases was not investigated.

Activity of compound **10** in rat brain tissue highlights the potential to use this new class of allosteric sGC inhibitors to study the role of the NO—sGC—cGMP signalling pathway in the brain. Reducing amounts of cGMP in cells would have implications in downstream signalling proteins, such as cGMP-dependent protein kinases and phosphodiesterases.^35^

## 4 Conclusion

We have identified a new type of small molecule inhibitors of sGC, which are thought to be the first class to act through allosteric regulation of the enzyme catalytic domain, rather than oxidation of the haem or through the purine p-site.^36^ It is possible that the inhibitors presented bind to the catalytic domain of sGC inducing a conformational change, or ‘locking’ the enzyme in a basal conformation, that is not favourable to activation by NO or GTP binding. The inhibitor also inhibits particulate GC but not the related adenylate cyclase. Compound **10** (**D12**), with an IC_50_ and *K*_D_ in the micromolar range, may be used as a new tool to inhibit the NO—sGC—cGMP signalling pathway, and further study its implications and regulatory functions in the brain.

## 5 Experimentals

Full-length human recombinant guanylyl cyclase (soluble) and bovine lung guanylyl cyclase (soluble) were obtained from Enzo lifesciences (catalogue numbers ALX-201-177 and ALX-202-039-C005). Biacore consumables were purchased from GE Healthcare (UK), including buffer stock solutions. All other reagents used were obtained from Sigma. Starting materials were either commercially available or synthesized according to methods reported in the literature. ^1^H and ^13^C NMR spectra were recorded on a Bruker AMX-300 or a Bruker AMX-500 spectrometer. Chemical shifts are reported as ppm relative to TMS internal standard. Mass spectra were recorded on a Fisons VG70-SE spectrometer (EI, FAB) or an Agilent 6100 Series LC-mass spectrometer using C-18 or C-4 columns. Microwave reactions were carried out using a CEM Discover microwave.

### 5.1 Surface plasmon resonance

Surface plasmon resonance (SPR) experiments were performed with a Biacore T200 instrument at 25 °C. Data processing and analysis were performed using BIAevaluation software and Scrubber2. All sensorgrams were double referenced by subtracting the response in a reference flowcell and a blank sample. Materials and methods used were the same as previously published.^24^

### 5.2 Soluble guanylate cyclase assay

In general, bovine lung soluble guanylyl cyclase (5 ng/ml) was incubated with 50 mM Tris, 0.3 mM MgCl, 100 μM EGTA, 0.045% BSA, and 1000 units/mL SOD at pH 7.4 and 1 mM MgGTP at 37 °C. Compounds were added at 100 μM prior to adding the NO donor DEA/NO (30 nM) or clamped NO concentrations. cGMP generation was allowed to proceed for two minutes, after which aliquots were withdrawn and inactivated by boiling in 50 mM Tris, 4 mM EDTA buffer. cGMP formation was measured by a standard radioimmunoassay.

### 5.3 Particulate guanylate cyclase assay

Purified rat lung membranes (20 μg protein/200 μL) were incubated in Tris buffer with 10 mM phosphocreatine, 10 units/mL creatine phosphokinase, and 10 μM ODQ to inhibit NO-activated GC. The reaction took place with 0.3 mM MgCl, 0.5 mM MgATP, and 1 mM MgGTP, at 37 °C. The compound was added and 1 μM ANP used to activate pGC. cGMP formation was allowed to proceed for 10.5–12.5 min and was subsequently measured by radioimmunoassay.

### 5.4 cGMP measurement in rat cerebellar slices

Brain slices methods were conducted as described in detail.^37,38^ Briefly, 0.4 mm thick sagittal cerebellar slices from 10-day-old animals were cut using a McIlwain tissue chopper. Experiments were carried out in the presence of the broad spectrum phosphodiesterase inhibitor IBMX (1 mM; 10 min preincubation) and, when appropriate, antagonists were added 10 min prior to IBMX. Following treatment, brain slices were inactivated in boiling Tris–HCl buffer (50 mM, pH 7.5) containing EDTA (4 mM) and homogenised by sonication after which aliquots were removed for measurement of protein (bicinchoninic acid method) and cGMP (radioimmunoassay).

### 5.5 Virtual screening

Similarity searching using MACCS fingerprints at 85% and 75% Tanimoto similarity was performed in 2007 using the following databases: Abinitio, Acros, Actimol, Akos/Akl, Akox/Akx, Akos/Owh, Asinex, Chembridge, Chemstar, LifeChemicals, MoscowMedChemLabs, Pharmeks, Sigma, SPECS, Vitas/Dah, Vitas/Stk. The available structures were selected by hand based upon diversity, molecular size, and apparent availability.

### 5.6 Molecular modelling

Docking was performed using the software Gold 5.1. The pdb file (3uvj) was imported and analysed, and missing residue side chains were added to the structure. The binding site was selected by importing a list of residues correspondent to the allosteric binding site. The fitness function selected was GOLDSCORE and the genetic algorithm search was performed at medium speed. Results were visualised in CCG MOE software.

### 5.7 Synthesis

#### 5.7.1 General synthesis of 2,3-dichloroquinoxalines **12**

A solution of oxalic acid in 4 N HCl (5 mL) was added to a stirring solution of the diamine (1:1) in 4 N HCl (15 mL) and the reaction mixture was refluxed for 2 h. The solution was cooled to room temperature and the precipitate filtered by suction, washed with water and freeze-dried. The resulting 2,3-dihydroxyquinoxaline **11** was dissolved in thionyl chloride (5 mL). A catalytic amount of *N*,*N*-dimethylformamide (0.01 equiv) was added to the solution which was stirred under reflux for 2 h. The reaction mixture was cooled to room temperature and concentrated under vacuum. Water (10 mL) was added to the solid on an ice bath and the slurry was stirred for 30 min at room temperature, after which the solid was filtered and washed with water. The precipitate was taken up in a minimum amount of DCM and filtered through a silica column and eluted with DCM.

#### 5.7.2 2,3-Dichloro-*N*-methylquinoxaline-6-carboxamide **13**

Methylamine solution (33 wt % in absolute ethanol, 256 μL) was added to DCM (3 mL) on ice and the resulting mixture was added to a solution of 2,3-dichloroquinoxaline-6-carbonyl chloride (261 mg, 1 mmol) in DCM (3 mL) whilst stirring at room temperature. The mixture was stirred at room temperature overnight after which it was diluted with DCM (10 mL) and washed with saturated sodium bicarbonate (3 × 10 mL) and brine (3 × 10 mL). The organics were dried over MgSO_4_, filtered, and the solved evaporated. The crude compound was purified by flash column chromatography using a gradient of 30–60% ethyl acetate in cyclohexane to yield the named compound as a white powder (89 mg, 35%). ^1^H NMR (500 MHz, *d*_6_-DMSO) *δ*: 8.84 (s, 1H, N*H*), 8.48 (s, 1H, Ar*H*), 8.29 (d, *J* = 8.6 Hz, 1H, Ar*H*), 8.14 (d, *J* = 8.6 Hz, 1H, Ar*H*), 2.85 (d, *J* = 3.7 Hz, 3H, C*H*_3_). ^13^C NMR (126 MHz, *d*_6_-DMSO) *δ*: 165.0 (Ar*C*), 146.0 (Ar*C*), 145.6 (Ar*C*), 141.1 (Ar*C*), 139.6 (Ar*C*), 136.8 (Ar*C*), 129.9 (Ar*C*H), 128.1 (Ar*C*H), 126.6 (Ar*C*H), 26.5 (*C*H_3_). MS-EI (*m*/*z*): [M] calculated for C_10_H_7_Cl_2_N_3_O, 256.089; found 255.90.

#### 5.7.3 General synthesis of compounds **10** and **14**–**29**

The quinoxaline and aniline (1:4) were transferred into a 10 mL microwave vial containing a stirrer bar and NMP (1 mL). The mixture was stirred at 160 °C for 5 min under microwave irradiation. The residue was taken up in water (5 mL) and extracted with ethyl acetate (3 × 5 mL). The organics were washed with water (3 × 5 mL) and brine (3 × 5 mL), dried over Mg_2_SO_4_, filtered and concentrated in vacuo. The final compound was purified by flash column chromatography.

##### 5.7.3.1 4,4′-((6-Nitroquinoxaline-2,3-diyl)bis(azanediyl))diphenol **10**

^1^H NMR (500 MHz, *d*_6_-DMSO) *δ*: 9.37 (s, 1H, O*H*), 9.31 (s, 1H, O*H*), 9.26 (s, 1H, N*H*), 9.07 (s, 1H, N*H*), 8.18 (d, *J* = 2.5 Hz, 1H, Ar*H*), 8.01 (dd, *J* = 8.9, 2.5 Hz, 1H, Ar*H*), 7.64 (dd, *J* = 10.9, 8.9 Hz, 4H, 4 × Ar*H*), 7.51 (d, *J* = 8.9 Hz, 1H, Ar*H*), 6.83 (d, *J* = 8.9 Hz, 4H, 4 × Ar*H*). ^13^C NMR (126 MHz, *d*_6_-DMSO) *δ* 154.2 (Ar*C*), 153.8 (Ar*C*), 143.2 (Ar*C*), 142.6 (Ar*C*), 141.5 (Ar*C*), 140.6 (Ar*C*), 135.3 (Ar*C*), 130.7 (Ar*C*), 130.3 (Ar*C*), 125.4 (Ar*C*H), 123.7 (Ar*C*H), 123.2 (Ar*C*H), 120.1 (Ar*C*H), 118.6 (Ar*C*H), 115.5 (Ar*C*H), 115.2 (Ar*C*H). HRMS-ES (*m*/*z*): [M+H]^+^ calculated for C_20_H_16_N_5_O_4_, 390.1202; found, 390.1205.

##### 5.7.3.2 3,3′-((6-Nitroquinoxaline-2,3-diyl)bis(azanediyl))diphenol **14**

^1^H NMR (500 MHz, *d*_6_-DMSO) *δ*: 9.50 (s, 1H, O*H*), 9.48 (s, 1H, O*H*), 9.41 (s, 1H, N*H*), 9.24 (s, 1H, N*H*), 8.32 (d, *J* = 2.6 Hz, 1H, Ar*H*), 8.11 (dd, *J* = 9.0, 2.6 Hz, 1H, Ar*H*), 7.66 (d, *J* = 2.0 Hz, 1H, Ar*H*), 7.64 (d, *J* = 9.0 Hz, 1H, Ar*H*), 7.51 (t, *J* = 7.3 Hz, 1H, Ar*H*), 7.27 (dd, *J* = 18.4, 8.0 Hz, 2H, 2 × Ar*H*), 7.22–7.16 (m, 2H, 2 × Ar*H*), 6.54 (ddd, *J* = 17.0, 8.0, 1.5 Hz, 2H, 2 × Ar*H*). ^13^C NMR (126 MHz, *d*_6_-DMSO) *δ*: 155.89, 155.55, 143.29, 143.23, 142.52, 141.43, 135.29, 132.27, 131.87, 125.53, 123.46, 122.95, 120.18, 118.80, 113.95, 55.25, 55.22. HRMS-ES (*m*/*z*): [M+H]^+^ calculated for C_22_H_16_N_5_O_4_, 390.1202; found, 390.1189.

##### 5.7.3.3 *N*2,*N*3-Bis(4-methoxyphenyl)-6-nitroquinoxaline-2,3-diamine **15**

^1^H NMR (500 MHz, *d*_6_-DMSO) *δ*: 9.39 (s, 1H, N*H*), 9.20 (s, 1H, N*H*), 8.21 (d, *J* = 2.6 Hz, 1H, Ar*H*), 8.04 (dd, *J* = 8.9, 2.6 Hz, 1H, Ar*H*), 7.82–7.74 (m, 4H, Ar*H*), 7.54 (d, *J* = 8.9 Hz, 1H, Ar*H*), 7.05–6.99 (m, 4H, Ar*H*), 3.78 (s, 6H, 2 × O*C*H_3_). ^13^C NMR (126 MHz, *d*_6_-DMSO) *δ* 155.9 (Ar*C*), 155.6 (Ar*C*), 143.3 (Ar*C*), 143.2 (Ar*C*), 142.5 (Ar*C*), 141.4 (Ar*C*), 135.3 (Ar*C*), 132.3 (Ar*C*), 131.9 (Ar*C*), 125.5 (Ar*C*H), 123.5 (Ar*C*H), 122.9 (Ar*C*H), 120.2 (Ar*C*H), 118.8 (Ar*C*H), 113.9 (Ar*C*H), 55.3 (O*C*H_3_), 55.2 (O*C*H_3_). HRMS-ES (*m*/*z*): [M+H]^+^ calculated for C_22_H_20_N_5_O_4_, 418.1515; found, 418.1531.

##### 5.7.3.4 6-Nitro-*N*2,*N*3-diphenylquinoxaline-2,3-diamine **16**

^1^H NMR (500 MHz, *d*_6_-DMSO) *δ*: 9.51 (s, 1H, N*H*), 9.34 (s, 1H, N*H*), 8.27 (d, *J* = 2.2 Hz, 1H, Ar*H*), 8.07 (dd, *J* = 8.8, 2.2 Hz, 1H, Ar*H*), 7.95–7.85 (m, 4H, 4 × Ar*H*), 7.62 (d, *J* = 8.8 Hz, 1H, Ar*H*), 7.47–7.39 (m, 4H, 4 × Ar*H*), 7.20–7.09 (m, 2H, 2 × Ar*H*). ^13^C NMR (126 MHz, *d*_6_-DMSO) *δ*: 143.7 (Ar*C*), 143.1 (Ar*C*), 142.4 (Ar*C*), 141.2 (Ar*C*), 139.4 (Ar*C*), 139.1 (Ar*C*), 135.2 (Ar*C*), 128.8 (Ar*C*H), 125.9 (Ar*C*H), 123.9 (Ar*C*H), 123.4 (Ar*C*H), 121.6 (Ar*C*H), 121.1 (Ar*C*H), 120.5 (Ar*C*H), 119.2 (Ar*C*H). HRMS-ES (*m*/*z*): [M+H]^+^ calculated for C_20_H_16_N_5_O_2_, 358.1304; found, 358.1305.

##### 5.7.3.5 *N*,*N*′-(((6-Nitroquinoxaline-2,3-diyl)bis(azanediyl))bis(4,1-phenylene))diacetamide **17**

^1^H NMR (500 MHz, *d*_6_-DMSO) *δ*: 9.97 (s, 1H, N*H*), 9.96 (s, 1H, N*H*), 9.52 (s, 1H, N*H*), 9.33 (s, 1H, N*H*), 8.28 (d, *J* = 2.4 Hz, 1H, Ar*H*), 8.06 (dd, *J* = 8.9, 2.4 Hz, 1H, Ar*H*), 7.83 (dd, *J* = 13.6, 8.9 Hz, 4H, 4 × Ar*H*), 7.67–7.57 (m, 5H, 5 × Ar*H*), 2.05 (d, *J* = 4.9 Hz, 6H, 6 × Ar*H*). ^13^C NMR (126 MHz, *d*_6_-DMSO) *δ*: 168.1 (Ar-NH-*C*O-CH_3_), 168.0 (Ar-NH-*C*O-CH_3_), 143.5 (Ar*C*), 143.1 (Ar*C*), 142.3 (Ar*C*), 141.3 (Ar*C*), 135.5 (Ar*C*), 135.3 (Ar*C*), 134.5 (Ar*C*), 134.1 (Ar*C*), 125.7 (Ar*C*H), 122.1 (Ar*C*H), 121.6 (Ar*C*H), 120.4 (Ar*C*H), 119.4 (Ar*C*H), 119.3 (Ar*C*H), 118.9 (Ar*C*H), 23.9 (Ar-NH-CO-*C*H_3_). HRMS-ES (*m*/*z*): [M+H]^+^ calculated for C_24_H_22_N_7_O_4_, 472.1733; found, 472.1750.

##### 5.7.3.6 *N*,*N*′-(((6-Nitroquinoxaline-2,3-diyl)bis(azanediyl))bis(3,1-phenylene))diacetamide **18**

^1^H NMR (500 MHz, *d*_6_-DMSO) *δ* 10.02 (s, 2H, 2 × N*H*), 9.62 (s, 1H, N*H*), 9.44 (s, 1H, N*H*), 8.46–8.32 (m, 3H, 3 × Ar*H*), 8.12 (dd, *J* = 8.8, 2.0 Hz, 1H, Ar*H*), 7.71–7.60 (m, 3H, Ar*H*), 7.36–7.25 (m, 5H, 5 × Ar*H*), 2.09 (s, 3H, CH_3_), 2.08 (s, 3H, CH_3_). ^13^C NMR (126 MHz, *d*_6_-DMSO) *δ* 168.4 (Ar-NH*C*OCH_3_), 139.7 (Ar*C*), 135.1 (Ar*C*), 128.8 (Ar*C*H), 125.9 (Ar*C*H), 120.6 (Ar*C*), 119.2 (Ar*C*H), 116.3 (Ar*C*H), 115.8 (Ar*C*H), 114.5 (Ar*C*H), 114.1 (Ar*C*H), 112.2 (Ar*C*H), 111.6 (Ar*C*), 109.1 (Ar*C*), 24.1 (Ar-NHCO*C*H_3_). HRMS-ES (*m*/*z*): [M+H]^+^ calculated for C_24_H_22_N_7_O_4_, 472.1733; found, 472.1733.

##### 5.7.3.7 *N*2,*N*3-Bis(4-fluorophenyl)-6-nitroquinoxaline-2,3-diamine **19**

^1^H NMR (500 MHz, *d*_6_-DMSO) *δ*: 9.52 (s, 1H), 9.36 (s, 1H), 8.27 (d, *J* = 2.4 Hz, 1H), 8.07 (dd, *J* = 8.9, 2.4 Hz, 1H), 7.92 (ddd, *J* = 13.8, 8.9, 5.0 Hz, 4H), 7.60 (d, *J* = 8.9 Hz, 1H), 7.31–7.23 (m, 4H). HRMS-ES (*m*/*z*): [M+H] calculated for C_20_H_14_N_5_O_3_F_2_, 394.1116; found, 394.1123.

##### 5.7.3.8 4,4′-(Quinoxaline-2,3-diylbis(azanediyl))diphenol **20**

^1^H NMR (500 MHz, *d*_6_-DMSO) *δ* 9.21 (s, 2H, 2 × O*H*), 8.71 (s, 2H, 2 × N*H*), 7.61 (d, *J* = 8.7 Hz, 4H, 4 × Ar*H*), 7.42 (dt, *J* = 7.3, 3.4 Hz, 2H, 2 × Ar*H*), 7.23 (dd, *J* = 6.1, 3.4 Hz, 2H, 2 × Ar*H*), 6.79 (d, *J* = 8.7 Hz, 4H, 4 × Ar*H*). ^13^C NMR (126 MHz, *d*_6_-DMSO) *δ* 153.2 (Ar*C*), 141.4 (Ar*C*), 136.2 (Ar*C*), 131.5 (Ar*C*), 125.0 (Ar*C*H), 124.4 (Ar*C*H), 122.8 (Ar*C*H), 115.1 (Ar*C*H). HRMS-ES (*m*/*z*): [M+H]^+^ calculated for C_20_H_17_N_4_O_2_, 345.1352; found, 345.1347.

##### 5.7.3.9 4,4′-((6-(Trifluoromethyl)quinoxaline-2,3-diyl)bis(azanediyl))diphenol **21**

^1^H NMR (500 MHz, *d*_6_-DMSO) *δ*: 9.34 (s, 1H, O*H*), 9.32 (s, 1H, O*H*), 9.03 (s, 1H, N*H*), 8.96 (s, 1H, N*H*), 7.68 (s, 1H, Ar*H*), 7.65–7.59 (m, 4H, 4 × Ar*H*), 7.55 (d, *J* = 8.5 Hz, 1H, Ar*H*), 7.47 (dd, *J* = 8.5, 1.8 Hz, 1H, Ar*H*), 6.84–6.78 (m, 4H, 4 × Ar*H*). ^13^C NMR (126 MHz, *d*_6_-DMSO) *δ*: 170.3 (Ar*C*), 153.8 (Ar*C*), 153.6 (Ar*C*), 142.8 (Ar*C*), 142.3 (Ar*C*), 138.7 (Ar*C*), 135.7 (Ar*C*), 131.0 (Ar*C*), 130.8 (Ar*C*), 125.8 (Ar*C*H), 123.4 (Ar*C*H), 123.2 (Ar*C*H), 121.9 (Ar*C*H), 119.9 (Ar*C*H), 115.2 (Ar*C*H). HRMS-CI (*m*/*z*): [M+H]^+^ calculated for C_21_H_16_F_3_N_4_O_2_, 413.12199; found, 413.120133.

##### 5.7.3.10 3,3′-((6-(Trifluoromethyl)quinoxaline-2,3-diyl)bis(azanediyl))diphenol **22**

^1^H NMR (500 MHz, *d*_6_-DMSO) *δ*: 9.49 (s, 1H, O*H*), 9.46 (s, 1H, O*H*), 9.18 (s, 1H, N*H*), 9.12 (s, 1H, N*H*), 7.81 (s, 1H, Ar*H*), 7.67 (d, *J* = 8.4 Hz, 1H, Ar*H*), 7.61–7.56 (m, 2H, 2 × Ar*H*), 7.50 (d, *J* = 2.5 Hz, 1H, Ar*H*), 7.26 (dd, *J* = 11.6, 9.0 Hz, 2H, 2 × Ar*H*), 7.18 (td, *J* = 8.4, 2.5 Hz, 2H, 2 × Ar*H*), 6.57–6.46 (m, 2H, 2 × Ar*H*). ^13^C NMR (126 MHz, *d*_6_-DMSO) *δ*: 157.6 (Ar*C*), 142.5 (Ar*C*), 142.0 (Ar*C*), 140.7 (Ar*C*), 138.5 (Ar*C*), 135.5 (Ar*C*), 129.3 (Ar*C*H), 126.2 (Ar*C*H), 123.5 (Ar*C*), 122.3 (Ar*C*H), 120.6 (Ar*C*), 112.0 (Ar*C*H), 111.7 (Ar*C*H), 110.5 (Ar*C*H), 110.2 (Ar*C*H), 108.3 (Ar*C*H), 108.0 (Ar*C*H). HRMS-CI (*m*/*z*): [M+H]^+^ calculated for C_21_H_16_F_3_N_4_O_2_, 413.12199; found, 413.121732.

##### 5.7.3.11 2,3-Bis((4-hydroxyphenyl)amino)quinoxaline-6-carbonitrile **23**

^1^H NMR (500 MHz, *d*_6_-DMSO) *δ*: 9.76 (s, 1H, O*H*), 9.73 (s, 1H, O*H*), 9.56 (s, 1H, N*H*), 9.43 (s, 1H, N*H*), 8.25 (s, 1H, Ar*H*), 8.04 (dt, *J* = 8.8, 3.2 Hz, 4H, 4 × Ar*H*), 7.93 (ddd, *J* = 11.1, 8.3, 2.3 Hz, 2H, 2 × Ar*H*), 7.24 (dt, *J* = 8.8, 3.2 Hz, 4H, 4 × Ar*H*). ^13^C NMR (126 MHz, *d*_6_-DMSO) *δ*: 153.9 (Ar*C*), 153.7 (Ar*C*), 142.9 (Ar*C*), 142.3 (Ar*C*), 139.6 (Ar*C*), 136.0 (Ar*C*), 130.8 (Ar*C*), 130.5 (Ar*C*), 129.3 (Ar*C*H), 126.5 (Ar*C*H), 125.9 (Ar*C*H), 123.6 (Ar*C*H), 123.2 (Ar*C*H), 119.5 (ArC-*C*N), 115.19 (Ar*C*H), 115.17 (Ar*C*H), 105.6 (ArC-*C*N). HRMS-CI (*m*/*z*): [M+H]^+^ calculated for C_21_H_16_N_5_O_2_, 370.13040; found, 370.129541.

##### 5.7.3.12 2,3-Bis((3-hydroxyphenyl)amino)quinoxaline-6-carbonitrile **24**

^1^H NMR (500 MHz, *d*_6_-DMSO) *δ*: 9.47 (s, 1H, O*H*), 9.42 (s, 1H, O*H*), 9.27 (s, 1H, N*H*), 9.16 (s, 1H, N*H*), 7.93 (d, *J* = 1.7 Hz, 1H, Ar*H*), 7.66–7.57 (m, 2H, 2 × Ar*H*), 7.53 (s, 1H, Ar*H*), 7.49 (s, 1H, Ar*H*), 7.30–7.24 (m, 2H, 2 × Ar*H*), 7.18 (dd, *J* = 14.3, 8.0 Hz, 2H, 2 × Ar*H*), 6.57–6.47 (m, 2H, 2 × Ar*H*). ^13^C NMR (126 MHz, *d*_6_-DMSO) *δ*: 157.6 (Ar*C*), 142.6 (Ar*C*), 142.0 (Ar*C*), 140.6 (Ar*C*), 140.3 (Ar*C*), 139.3 (Ar*C*), 135.8 (Ar*C*), 129.7 (Ar*C*H), 129.3 (Ar*C*H), 127.0 (Ar*C*H), 126.3 (Ar*C*H), 119.3 (Ar-*C*), 112.1 (Ar*C*H), 111.8 (Ar*C*H), 110.8 (Ar*C*H), 110.4 (Ar*C*H), 108.4 (Ar*C*H), 108.1 (Ar*C*H), 106.4 (ArC-*C*N). HRMS-CI (*m*/*z*): [M+H]^+^ calculated for C_21_H_16_N_5_O_2_, 370.13040; found, 370.129114.

##### 5.7.3.13 4,4′-((6-Chloroquinoxaline-2,3-diyl)bis(azanediyl))diphenol **25**

^1^H NMR (500 MHz, *d*_6_-DMSO) *δ*: 9.24 (s, 1H), 9.23 (s, 1H), 8.87 (s, 1H), 8.81 (s, 1H), 7.65–7.55 (m, 4H), 7.45–7.37 (m, 2H), 7.22 (dt, *J* = 8.6, 2.1 Hz, 1H), 6.83–6.76 (m, 4H). ^13^C NMR (126 MHz, *d*_6_-DMSO) *δ*: 153.51 (Ar*C*), 153.45 (Ar*C*), 141.97 (Ar*C*), 141.61 (Ar*C*), 137.03 (Ar*C*), 134.97 (Ar*C*), 131.18 (Ar*C*), 131.09 (Ar*C*), 128.04 (Ar*C*), 126.35 (Ar*C*H), 124.25 (Ar*C*H), 123.73 (Ar*C*H), 123.07 (Ar*C*H), 123.04 (Ar*C*H), 115.14 (Ar*C*H). HRMS-ES (*m*/*z*): [M+H] calculated for C_20_H_16_N_4_O_2_Cl, 379.0962; found, 379.0927.

##### 5.7.3.14 3,3′-((6-Chloroquinoxaline-2,3-diyl)bis(azanediyl))diphenol **26**

^1^H NMR (500 MHz, *d*_6_-DMSO) *δ*: 9.39 (s, 2H), 9.03 (s, 1H), 8.98 (s, 1H), 7.58–7.49 (m, 3H), 7.48 (s, 1H), 7.33 (dd, *J* = 8.7, 2.3 Hz, 1H), 7.23 (s, 2H), 7.16 (td, *J* = 8.0, 1.7 Hz, 2H), 6.50 (d, *J* = 8.0 Hz, 2H). ^13^C NMR (126 MHz, *d*_6_-DMSO) *δ*: 157.58 (Ar*C*), 141.66 (Ar*C*), 141.25 (Ar*C*), 140.92 (Ar*C*), 140.83 (Ar*C*), 136.82 (Ar*C*), 134.79 (Ar*C*), 129.22 (Ar*C*H), 128.78 (Ar*C*), 126.72 (Ar*C*H), 125.03 (Ar*C*H), 124.05 (Ar*C*H), 111.55 (Ar*C*H), 110.06 (Ar*C*H), 107.83 (Ar*C*H). HRMS-ES (*m*/*z*): [M+H] calculated for C_20_H_16_N_4_O_2_Cl, 379.0962; found, 379.0940.

##### 5.7.3.15 2,3-Bis((4-hydroxyphenyl)amino)-*N*-methylquinoxaline-6-carboxamide **27**

^1^H NMR (500 MHz, *d*_6_-DMSO) *δ* 9.26 (s, 1H, O*H*), 9.25 (s, 1H, O*H*), 8.89 (s, 1H, Ar-N*H*), 8.82 (s, 1H, Ar-N*H*), 8.44 (q, *J* = 4.5 Hz, 1H, Ar-CON*H*CH_3_), 7.94 (d, *J* = 2.0 Hz, 1H, Ar*H*), 7.71 (dd, *J* = 8.4, 2.0 Hz, 1H, Ar*H*), 7.63 (d, *J* = 8.4 Hz, 4H, 4 × Ar*H*), 7.43 (d, *J* = 8.4 Hz, 1H, Ar*H*), 6.86–6.77 (m, 4H, 4 × Ar*H*), 2.77 (d, *J* = 4.5 Hz, 3H, Ar-CONHC*H*_3_). ^13^C NMR (126 MHz, *d*_6_-DMSO) *δ* 166.5 (Ar-*C*ONHCH_3_), 153.5 (Ar*C*), 153.4 (Ar*C*), 142.2 (Ar*C*), 141.9 (Ar*C*), 138.2 (Ar*C*), 137.2 (Ar*C*), 135.4 (Ar*C*), 131.3 (Ar*C*), 131.1 (Ar*C*), 130.0 (Ar*C*H), 124.6 (Ar*C*H), 123.8 (Ar*C*H), 123.1 (Ar*C*H), 115.1 (Ar*C*H), 26.3 (Ar-CONH*C*H_3_). HRMS-EI (*m*/*z*): [M] calculated for C_22_H_19_N_5_O_3_, 401.1488; found, 401.1481.

##### 5.7.3.16 6,7-Bis((3-hydroxyphenyl)amino)-*N*-methyl-2-naphthamide **28**

^1^H NMR (500 MHz, *d*_6_-DMSO) *δ* 9.42 (s, 1H, O*H*), 9.41 (s, 1H, O*H*), 9.04 (s, 1H, N*H*), 8.98 (s, 1H, N*H*), 8.51 (d, *J* = 4.4 Hz, 1H, Ar-CON*H*CH_3_), 8.06 (s, 1H, ArC*H*), 7.80 (d, *J* = 8.4 Hz, 1H, ArC*H*), 7.55 (d, *J* = 8.4 Hz, 1H, ArC*H*), 7.50 (d, *J* = 12.0 Hz, 2H, 2 × ArC*H*), 7.28 (d, *J* = 8.0 Hz, 2H, 2 × ArC*H*), 7.17 (t, *J* = 8.0 Hz, 2H, 2 × ArC*H*), 6.50 (d, *J* = 8.0 Hz, 2H, 2 × ArC*H*), 2.80 (d, *J* = 4.4 Hz, 3H, Ar-CONHC*H*_3_). ^13^C NMR (126 MHz, *d*_6_-DMSO) *δ* 166.5 (Ar-*C*ONHCH_3_), 157.6 (Ar*C*), 157.6 (Ar*C*), 141.9 (Ar*C*), 141.6 (Ar*C*), 141.0 (Ar*C*), 140.9 (Ar*C*), 137.9 (Ar*C*), 135.3 (Ar*C*), 130.8 (Ar*C*H), 129.22 (Ar*C*H), 129.19 (Ar*C*H), 124.9 (Ar*C*), 124.3 (Ar*C*), 123.7 (Ar*C*), 111.7 (Ar*C*H), 111.6 (Ar*C*H), 110.2 (Ar*C*H), 110.0 (Ar*C*H), 108.0 (Ar*C*H), 107.9 (Ar*C*H), 26.3 (Ar-CONH*C*H_3_). HRMS-EI (*m*/*z*): [M] calculated for C_22_H_19_N_5_O_3_, 401.1488; found, 401.1479.

#### 5.7.4 4,4′-((6-Aminoquinoxaline-2,3-diyl)bis(azanediyl))diphenol **29**

Tin(II) chloride dihydrate (112.5 mg, 0.5 mmol) was added to a solution of 4,4′-((6-nitroquinoxaline-2,3-diyl)bis(azanediyl))diphenol **10** (39 mg, 0.1 mmol) in ethanol (15 mL) and the resulting mixture was brought to boil. A solution of sodium borohydride (18.9 mg, 0.5 mmol) in ethanol (5 mL) was added to the boiling mixture dropwise and refluxed for 1.5 h. Upon cooling, water (10 mL) was added. Sodium hydroxide was added until neutralisation of the solution. The ethanol was evaporated from the mixture and an extraction with ethyl acetate (20 mL) was performed. The organics were washed with brine (3 × 10 mL) and dried over magnesium sulfate, filtered and the solvent evaporated under reduced pressure. The crude material was purified by flash column chromatography using a gradient of 50–80% ethyl acetate in cyclohexane to give the named compound (22.0 mg, 61%). ^1^H NMR (500 MHz, *d*_6_-DMSO) *δ*: 9.13 (s, 1H, O*H*), 9.06 (s, 1H, O*H*), 8.47 (s, 1H, N*H*), 8.23 (s, 1H, N*H*), 7.60 (d, *J* = 8.9 Hz, 2H, 2 × Ar*H*), 7.53 (d, *J* = 8.9 Hz, 2H, 2 × Ar*H*), 7.15 (d, *J* = 8.6 Hz, 1H, Ar*H*), 6.75 (dd, *J* = 10.8, 8.9 Hz, 4H, 4 × Ar*H*), 6.64 (dd, *J* = 8.6, 2.4 Hz, 1H, Ar*H*), 6.58 (d, *J* = 2.4 Hz, 1H, Ar*H*), 5.06 (s, 2H, N*H*_2_). ^13^C NMR (126 MHz, *d*_6_-DMSO) *δ*: 152.8 (Ar*C*), 152.4 (Ar*C*), 146.3 (Ar*C*), 141.4 (Ar*C*), 138.5 (Ar*C*), 137.6 (Ar*C*), 132.5 (Ar*C*), 132.0 (Ar*C*), 128.4 (Ar*C*), 125.4 (Ar*C*H), 122.4 (Ar*C*H), 121.8 (Ar*C*H), 115.0 (Ar*C*H), 114.99 (Ar*C*H), 114.8 (Ar*C*H), 106.5 (Ar*C*H). HRMS-CI (*m*/*z*): [M+H]^+^ calculated for C_20_H_18_N_5_O_2_, 360.1460; found, 360.1451.

## Figures and Tables

**Table 1:** % inhibition of sGC and SPR binding level of synthesised analogues of compound **10** to sGCcat

**Figure:**
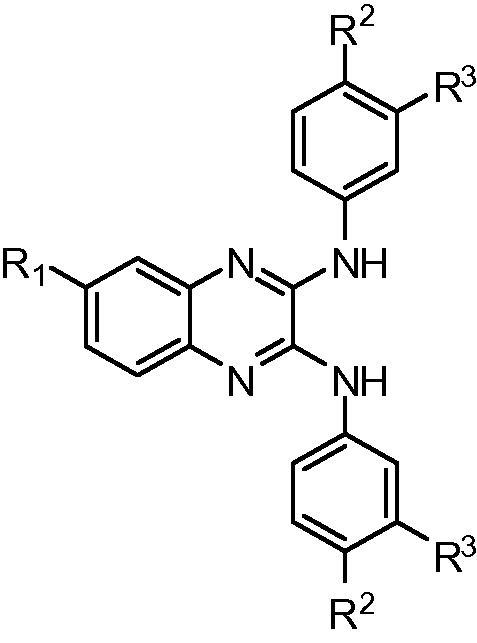

Compd number	R1	R2	R3	sGCcat (SPR) binding norm. resp. (RU)	sGC activity% inhibition at 100 μM
**10**	NO_2_	OH	H	14.4 ± 1.7	56.2 ± 22.4
**14**	NO_2_	H	OH	11.6 ± 0.8	41.4
**15**	NO_2_	OCH_3_	H	1.0 ± 0.3	—
**16**	NO_2_	H	H	2.6 ± 0.2	—
**17**	NO_2_	NHCOCH_3_	H	0.8 ± 0.3	—
**18**	NO_2_	H	NHCOCH_3_	3.0 ± 1.0	—
**19**	NO_2_	F	H	1.9 ± 1.8	—
**20**	H	OH	H	12.4 ± 0.1	—
**21**	CF_3_	OH	H	6.4 ± 0.5	44.4
**22**	CF_3_	H	OH	3.0 ± 0.5	21.1
**23**	CN	OH	H	4.5 ± 2	—
**24**	CN	H	OH	10.5 ± 0.5	—
**25**	Cl	OH	H	9.7 ± 0.5	—
**26**	Cl	H	OH	9.0 ± 0.4	—
**27**	CONHCH_3_	OH	H	1.1 ± 0.3	—
**28**	CONHCH_3_	H	OH	9.6 ± 0.3	—
**29**	NH_2_	OH	H	9.9 ± 0.2	—

**Table 2:** sGC activity of compound **10**

GC activator		Compound **10** IC_50_ (μM) ± SE	Hill slope
Cinaciguat	1 μM	37 ± 40	4
20 nM	25 ± 4	4

ANP	1 μM	25 ± 13	1
10 nM	21 ± 3	2
Basal	15 ± 2	2
